# Evaluation of the Temporal Muscle Thickness as an Independent Prognostic Biomarker in Patients with Primary Central Nervous System Lymphoma

**DOI:** 10.3390/cancers13030566

**Published:** 2021-02-02

**Authors:** Julia Furtner, Karl-Heinz Nenning, Thomas Roetzer, Johanna Gesperger, Lukas Seebrecht, Michael Weber, Astrid Grams, Stefan L. Leber, Franz Marhold, Camillo Sherif, Johannes Trenkler, Barbara Kiesel, Georg Widhalm, Ulrika Asenbaum, Ramona Woitek, Anna S. Berghoff, Daniela Prayer, Georg Langs, Matthias Preusser, Adelheid Wöhrer

**Affiliations:** 1 Department of Biomedical Imaging and Image-Guided Therapy, Medical University of Vienna, 1090 Vienna, Austria; michael.weber@meduniwien.ac.at (M.W.); ulrika.asenbaum@meduniwien.ac.at (U.A.); ramona.woitek@meduniwien.ac.at (R.W.); daniela.prayer@meduniwien.ac.at (D.P.); 2 Computational Imaging Research Lab, Department of Biomedical Imaging and Image-Guided Therapy, Medical University of Vienna, 1090 Vienna, Austria; karl-heinz.nenning@meduniwien.ac.at (K.-H.N.); georg.langs@meduniwien.ac.at (G.L.); 3 Division of Neuropathology and Neurochemistry, Department of Neurology, Medical University of Vienna, 1090 Vienna, Austria; Thomas.roetzer@meduniwien.ac.at (T.R.); johanna.gesperger@meduniwien.ac.at (J.G.); lukas.seebrecht@gmx.at (L.S.); adelheid.woehrer@meduniwien.ac.at (A.W.); 4 Department of Neuroradiology, Medical University of Innsbruck, 6020 Innsbruck, Austria; astrid.grams@i-med.ac.at; 5 Division of Neuroradiology, Vascular and Interventional Radiology, Department of Radiology, Medical University of Graz, 8036 Graz, Austria; stefan.leber@medunigraz.at; 6 Division of Neurosurgery, University Hospital St. Poelten, Karl Landsteiner University of Health Sciences, 3100 St. Poelten, Austria; franz.marhold@stpoelten.lknoe.at; 7 Department of Neurosurgery, Krankenanstalt Rudolfstiftung, 1030 Wien, Austria; camillo.sherif@gmail.com; 8 Institute of Neuroradiology, NeuromedCampus, Kepler University Hospital, Johannes Kepler Universität of Linz, 4020 Linz, Austria; Johannes.trenkler@kepleruniklinikum.at; 9 Department of Neurosurgery, Medical University of Vienna, 1090 Vienna, Austria; Barbara.kiesel@meduniwien.ac.at (B.K.); georg.widhalm@meduniwien.ac.at (G.W.); 10 Department of Medicine I, Division of Oncology, Medical University of Vienna, 1090 Vienna, Austria; anna.berghoff@meduniwien.ac.at (A.S.B.); matthias.preusser@meduniwien.ac.at (M.P.)

**Keywords:** primary central nervous system lymphoma, temporal muscle thickness, sarcopenia, prognostic parameter, overall survival

## Abstract

**Simple Summary:**

Primary central nervous system lymphoma (PCNSL) is a rare brain tumor with an exceedingly poor outcome. Although some of the established prognostic parameters in PCNSL patients, such as age, blood-related parameters, or the involvement of deep brain structures, are objectively evaluable, the information about the patient’s physical condition is still based on the subjective perception of the attending physician. The thickness of the temporal muscle has previously shown to be a biomarker of skeletal muscle quantity and quality, and thus be a potential parameter reflecting sarcopenia, which is a main feature of cancer-related cachexia and a well-known prognostic marker in various disease entities. In the current study we show that temporal muscle thickness is an independent and objectively assessable parameter for outcome prognostication in PCNSL patients and may facilitate the selection and stratification of patients for treatment options or clinical trials in the future.

**Abstract:**

In this study, we assessed the prognostic relevance of temporal muscle thickness (TMT), likely reflecting patient’s frailty, in patients with primary central nervous system lymphoma (PCNSL). In 128 newly diagnosed PCNSL patients TMT was analyzed on cranial magnetic resonance images. Predefined sex-specific TMT cutoff values were used to categorize the patient cohort. Survival analyses, using a log-rank test as well as Cox models adjusted for further prognostic parameters, were performed. The risk of death was significantly increased for PCNSL patients with reduced muscle thickness (hazard ratio of 3.189, 95% CI: 2–097–4.848, *p* < 0.001). Importantly, the results confirmed that TMT could be used as an independent prognostic marker upon multivariate Cox modeling (hazard ratio of 2.504, 95% CI: 1.608–3.911, *p* < 0.001) adjusting for sex, age at time of diagnosis, deep brain involvement of the PCNSL lesions, Eastern Cooperative Oncology Group (ECOG) performance status, and methotrexate-based chemotherapy. A TMT value below the sex-related cutoff value at the time of diagnosis is an independent adverse marker in patients with PCNSL. Thus, our results suggest the systematic inclusion of TMT in further translational and clinical studies designed to help validate its role as a prognostic biomarker.

## 1. Introduction

Primary cerebral nervous system lymphoma (PCNSL) is a devastating neoplasm of the brain, accounting for 3–4% of all brain tumors [1,2]. Despite the sustained efforts at therapeutic improvement, the prognosis of PCNSL patients remains poor [3,4]. Previous investigations have revealed that the outcome among various therapeutic strategies in PCNSL patients was attributed to a prognostic marker rather than to treatment efficacy [5]. This underlines the need for reliable and objectively assessable prognostic markers for accurate risk stratification for clinical trials as well as decision-making in the routine clinical setting. Most of the previously identified prognostic parameters in PCNSL patients, such as age, blood-related parameters, or the involvement of deep brain structures, are objectively evaluable [6]. However, the assessment of the patients’ clinical condition in particular is mainly based on the subjective rating of the attending physician, which leads to an increased inter-observer variability and a reduced accuracy of survival prediction [7,8]. Thus, objectively assessable parameters with regard to the evaluation of the patients’ frailty are needed in order to improve the prognostic assessment of PCNSL patients. An increasingly used procedure with which to objectively assess a patient’s physical condition is the estimation of their skeletal muscle mass, which is, together with reduced muscle function, referred to as sarcopenia, a main feature of cancer-related cachexia [9].

Sarcopenia has been previously shown to have a significant impact on patient prognosis in various cancer types [10,11,12,13,14,15]. In patients with extracranial tumors, a widely used technique to estimate skeletal muscle mass is the assessment of the skeletal muscle cross-sectional area on abdominal computed tomography (CT) scans at the level of the third lumbar vertebra. The standard tool with which to investigate the skeletal muscle function is the grip strength of the dominant hand. In particular, in brain tumor patients, neither CT images of the abdomen nor grip strength measurements are routinely performed. To obtain these examinations solely to assess sarcopenia would result in a prolonged clinical examination, increased radiation exposure, and additional healthcare costs. However, recently, temporal muscle thickness (TMT) has been suggested as a new biomarker with which to determine frailty, as it highly correlates with skeletal muscle mass as assessed by the use of calf circumference, arm muscle circumference, and the lumbar skeletal muscle cross-sectional area [16,17]. Moreover, TMT showed a high correlation with the grip strength of the dominant hand in healthy volunteers, as well as in patients with various neurological diseases [18]. These studies indicate that the estimation of skeletal muscle mass is not limited to lumbar muscles, but can also be determined on craniofacial muscles.

In the current study, we evaluated the prognostic value of TMT in PCNSL patients at the time of diagnosis to assess the importance of TMT as a prognostic parameter in this brain tumor entity.

## 2. Results

The retrospective study cohort consisted of 128 patients with newly diagnosed, treatment-naïve PCNSL. An overview of patient characteristics is given in Table 1.

All 128 included PCNSLs were of the diffuse large B-cell lymphoma (DLBCL) type. Ten of these patients suffered from an immunodeficiency, comprising nine drug-induced immunocompromised patients (e.g., after organ transplantation) and one HIV patient. Patients received either chemotherapy (*n* = 81), radiation therapy (*n* = 11), radiochemotherapy (*n* = 26), or best supportive care (*n* = 10). In addition, 26 patients were treated with rituximab (20 patients from the chemotherapy subgroup and six patients from the radiochemotherapy subgroup). However, due to the known prognostic relevance, only methotrexate-based chemotherapy was included in the multivariate Cox regression model to avoid any interdependencies between different therapy schemes.

For TMT measurements, exclusively T1-weighted contrast-enhanced magnetic resonance (MR) images without fat suppression were used. Due to the fact that the data were acquired in various different institutions over a large time range in order to compile a relatively large sample size of this rare disease entity, the MR sequences used for the TMT measurements were not identical with regard to their sequence parameters. However, in 91% (*n* = 117) of all patients, an isovoxel (1 × 1 × 1 mm) T1-weighted contrast-enhanced MR sequence was available for TMT assessment. Moreover, the MR examinations were performed on MR scanners with different field strengths (1.5 tesla in 59% of the patients (*n* = 76) and 3 tesla in 41% (*n* = 52), respectively). In 124 patients (97%), TMT measurements were available for both sides, whereas in four patients, TMT could be assessed only on one side due to post-interventional muscle alterations (biopsy, *n* = 2; previous craniotomy not related to the PCNSL, *n* = 2). The mean TMT value was 7 mm (range = 3.1 mm–10.5 mm) in male PCNSL patients and 5.4 mm (range 1.9 mm–8 mm) in female patients, resulting in an overall mean TMT of 6.3 mm. TMT values for female patients were significantly lower (*p* < 0.001; Student’s *t*-test).

**Figure 1:**
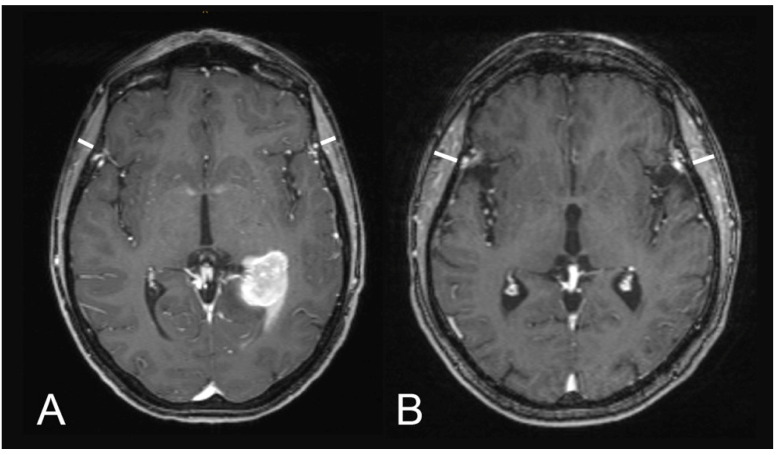
Illustration of TMT assessments on T1-weighted contrast-enhanced magnetic resonance images: (**A**) a 60-year-old male patient with an overall survival of one month (median TMT = 5.75 mm) and (**B**) a 51-year-old male patient with an overall survival of 73 months (median TMT = 8.1 mm).

Figure 1 illustrates examples of TMT measurements on T1-weighted contrast-enhanced MR images.

Previously defined sex-related cutoff values with regard to the diagnosis of sarcopenia 2.5 standard deviations (SD) below the mean TMT values of a normative reference (defined as healthy volunteers between 18 and 40 years old; male cutoff value ≤ 6.3 mm; female cutoff value ≤ 5.2 mm) were used to divide the patient cohorts, separated by sex, into two groups (male patients, *n* = 66, female patients, *n* = 62). Twenty-two male patients (33%) and 24 female patients (39%) showed mean TMT values below the sex-related cutoff values. The mean overall survival of patients below the TMT cutoff values was 9.4 months (SD: 2 months) compared to patients with a TMT above the cutoff values, which showed a mean overall survival of 54.2 months (SD: 6.2 months).

**Figure 2:**
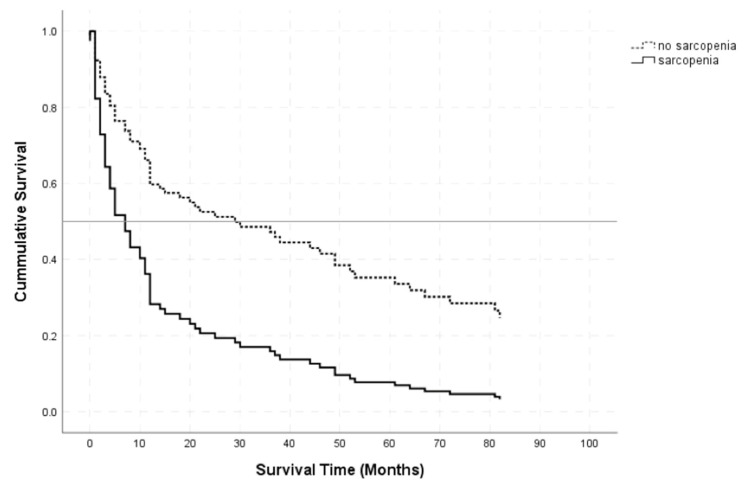
Kaplan–Meier survival curves for patients with TMT values below (black line) and above (dashed line) the sex-related cutoff values.

Kaplan–Meier curves separating patients with mean TMT values below (black line) and above (dashed line) the corresponding sex-related cutoff are visualized in Figure 2.

The log-rank test revealed a significant difference in overall survival between PCNSL patients below and above the sex-related cutoff vales (*p* < 0.001). A univariate Cox regression for overall survival as a function of the sex-related TMT cutoff resulted in an HR of 3.189 (95% CI: 2.097–4.848, *p* < 0.001) for sarcopenia.

**Figure 3:**
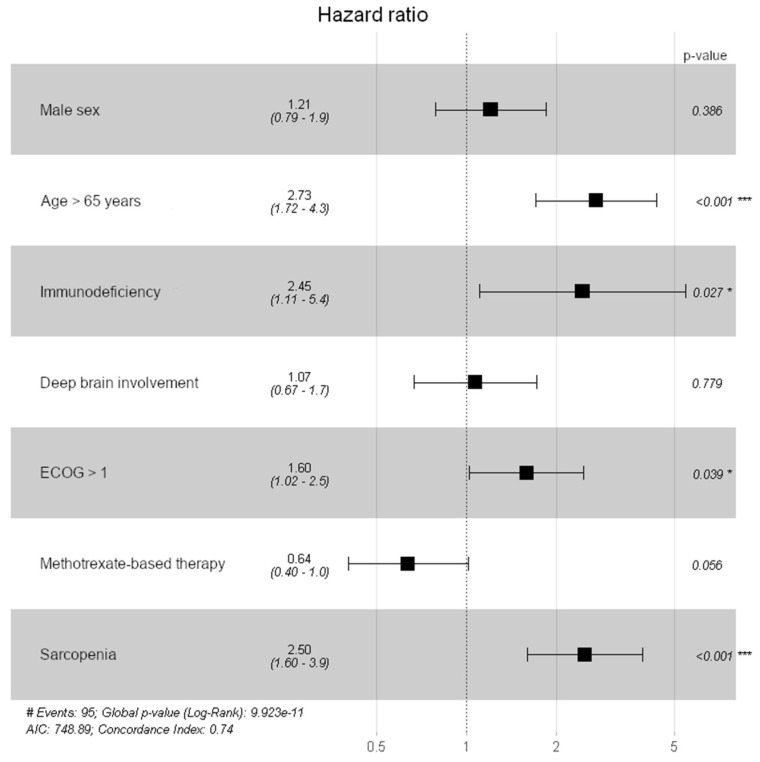
Forest plot to visualize the impact on all possible explanatory variables on overall survival (* *p* < 0.05; *** *p* < 0.001).

A multivariate Cox regression model for overall survival revealed significant results for TMT values ≤ the sex-related cutoff values (HR of 2.504, 95% CI: 1.608–3.911, *p* < 0.001), patient’s age ≥ 65 years (HR of 2.731, 95% CI: 1.717–4.344, *p* < 0.001), an ECOG performance status > 1 at the time of diagnosis (HR of 1.595, 95% CI: 1.025–2.483, *p* = 0.039), and immunodeficiency (HR of 2.453; 95% 1.108–5.43, *p* = 0.027), whereas male sex (HR of 1.209, 95% CI: 0.787–1.855, *p* = 0.386), deep brain involvement (HR of 1.070, 95% CI: 0.666–1.721, *p* = 0.779), and methotrexate-based chemotherapy (HR of 0.638, 95% CI: 0.402–1.012, *p* = 0.056) did not show a significant impact on overall survival. To visualize these results a forest blot overview of all other possible explanatory variables for overall survival is given in Figure 3.

## 3. Discussion

The current study revealed a strong prognostic impact of TMT with regard to overall survival in patients with PCNSL at the time of diagnosis. Patients with TMT values above the predefined, sex-related cutoff value (2.5 SD below the normative references; male TMT ≤ 6.3 mm; female TMT ≤ 5.2 mm) had a significantly longer overall survival (54.2 months; SD 6.2 months) compared to patients with a TMT below the cutoff value (9.4 months; SD 2 months; *p* < 0.001). Thus, the risk of death was shown to be significantly increased in patients with decreased sex-specific TMT values compared to patients with normal TMT values (univariable Cox regression HR: 3.189, 95% CI: 2.097–4.848, *p* < 0.001). The results of this study confirm and extend previous findings in glioblastoma, brain metastases, and aneurysmal subarachnoid hemorrhage patients in which TMT had already shown a strong association with overall survival [13,14,15,19,20,21,22].

The prognostic information of TMT in PCNSL patients was independent of other possible explanatory variables. Among sex, age at time of diagnosis, immunodeficiency, deep brain involvement of the PCNSL lesions, ECOG performance status, methotrexate-based chemotherapy, and TMT values a multivariable Cox regression analysis revealed a significant impact of patient’s age ≥ 65 years at the time of diagnosis (HR of 2.731, 95% CI: 1.717–4.344, *p* < 0.001), immunodeficiency (HR of 2.453; 95% 1.108–5.43, *p* = 0.027), ECOG performance status > 1 at time of diagnosis (HR of 1.595, 95% CI: 1.025–2.483, *p* = 0.039), TMT values below the sex-related cutoff values at the time of diagnosis (HR of 2.504, 95% CI: 1.608–3.911, *p* < 0.001), and a trend of methotrexate-based chemotherapy (HR of 0.638, 95% CI: 0.402–1.012, *p* = 0.056) on overall survival.

The patients in this study were stratified according to sex-related TMT cutoff values published previously [18]. Those sex related cutoff values were set 2.5 SD below the normative references (defined as healthy adults between 18–40 years old) as determined by the updated European Working Group on Sarcopenia in Older People (EWGSOP) recommendations [23]. Despite the high awareness of the relevance of a sarcopenia diagnosis due to the increased subjective but also socioeconomic burden of those patients, the assessment of the loss of skeletal muscle mass and strength is still not routinely available [24,25,26,27,28]. This may be due to the fact that the diagnosis of sarcopenia depends on additional examinations that result in prolonged clinical examinations, higher radiation doses for the patients, and additional health care costs. Thus, an objectively and quantitatively assessable tool can be easily integrated in the clinical setting and well suited to provide a rapid overview of the patient’s skeletal muscle status, which represents an unmet medical need, especially in patients with neurological disorders. In the current study, we validated the impact of these sex-related TMT cutoff values with regard to overall survival in PCNSL patients. Based on the results of our study, we suggest including TMT measurements, obtained from diagnostic MR images at the time of diagnosis, in the routine clinical workflow as an additional parameter reflecting a patient’s physical condition to establish real-life evidence for its biomarker potential. In patients who present TMT values below the sex-related cutoff values, further procedures should be initiated to rule out or confirm the diagnosis of sarcopenia as recommended in the EWGSOP guidelines [23]. Therefore, TMT values should be used to obtain an initial overview of a patient’s skeletal muscle mass and strength without replacing other diagnostic procedures. Further prospective clinical trials are necessary to validate the results of the current study and evaluate the impact of sarcopenia prevention or therapy on the clinical outcome of patients with PCNSL.

Besides the temporal muscle, other craniofacial muscles were also previously taken into consideration to estimate the skeletal muscle mass. Kilgour et al. assessed the cross-sectional area of the neck muscles at the midpoint level of C2 to evaluate the age-related loss of skeletal muscle mass on MR examinations of the brain [29]. The main limitation of this method is that in most cases, these muscles are incompletely depicted on routinely performed MRI examinations of the brain. Despite the relatively small muscle diameter, there are several reasons for the use of the temporal muscle as a prognostic parameter. It is one of the few craniofacial muscles that are depicted in their entirety on routine diagnostic MR images of the brain. This is particularly important for excluding muscle edema or atrophy in patients who have undergone a previous craniotomy or radiation therapy. Furthermore, the TMT measurements are associated with little expenditure of time, with an average duration of about 30 s per patient compared to other techniques. This makes the TMT assessment a suitable tool for the estimation of skeletal muscle mass and function that could be easily included in the routine clinical workflow. Moreover, previous studies could reveal an excellent inter-rater (same subject–same scanner–different reader) and intra-rater (same subject–same scanner–same reader) agreement (ICC > 0.9) each [15,18]. To ensure a high measurement accuracy the usage of isovoxel T1-weighted images is of utmost importance to reduce the partial volume effect. The use of T1-weighted images without fat saturation is crucial to optimize the visualization of the borders of the temporal muscle. Moreover, it is essential for a high intra-reader and inter-reader agreement to take precise measurements on magnified MR images and adhere strictly to the predefined anatomical landmarks (anterior commissure–posterior commissure line, level of the orbital roof and lateral sulcus). To reduce the potential influence of diseases of the temporo-mandibular joint or of the teeth, which may result in an asymmetry of the masticatory muscles, TMT measurements comprising both sides are used [30].

The major limitation of this study is the retrospective study design. Despite the large, national, multicenter patient cohort, prospective clinical trials are needed to evaluate the association between TMT with clinical frailty parameters. Moreover, the use of steroids by the patients of this study cohort was not taken into account, which could have had an impact on the thickness of the temporal muscle due to the known effect of steroids on muscle-wasting. However, TMT was assessed at the time of PCNSL diagnosis; thus, a prolonged exposure to corticosteroids seems unlikely.

Sarcopenia in cancer patients is considered a multifactorial event, particularly involving inflammatory and catabolic processes, and thus not solely reversible through nutritional diet. This makes it all the more important to identify patients at risk of sarcopenia by integrating TMT assessment in the daily workflow of patients with PCNSL in order to recognize the onset of skeletal muscle-mass loss and implement interventions to improve or at least delay the progression of this process. Another advantage of regular follow-up examinations is to be able to monitor the muscular status and specifically identify patients whose condition worsens over the course of their disease.

## 4. Materials and Methods

### 4.1. Patients

All newly diagnosed PCNSL patients between 2005 and 2018 were selected from the Austrian Brain Tumor Registry. MR images at time of diagnoses were retrospectively provided by the following institutions: Medical University of Vienna (*n* = 34), Kepler University Hospital Linz (*n* = 33), Paracelsus Private Medical University Salzburg (*n* = 3), Medical University of Innsbruck (*n*= 16), University Hospital of St. Poelten (*n* = 8), State Hospital of Klagenfurt (*n* = 15), Medical University of Graz (*n* = 12), and the Rudolfstiftung Hospital (*n* = 7). Related clinical data with potential prognostic value with regard to overall survival comprised sex, age, involvement of the deep brain area (defined as tumorous lesions that involve periventricular regions, the corpus callosum, the basal ganglia, the thalamus, the brain stem, or the cerebellum), Eastern Cooperative Oncology Group (ECOG) performance status, and individual patients’ treatment were retrieved by chart review. All PCNSL patients enrolled in this study were of the DLBCL type. The Epstein-barr virus (EBV) status was abstracted from pathology reports whenever available and positive in all 10 immunodeficiency associated lymphomas (*n* = 10).

The study was approved by the local ethics committee of the Medical University of Vienna (Vote EK 1140/2018).

### 4.2. Assessment of Temporal Muscle Thickness

We retrospectively analyzed TMT on routinely performed diagnostic MR images of the brain at the time of PCNSL diagnosis. The measurements were performed by a board-certified radiologist (JF) on axial contrast-enhanced T1-weighted MR images without fat-saturation, oriented parallel to the anterior commissure–posterior commissure line. TMT was assessed perpendicular to the long axis of the temporal muscle at the level of the orbital roof (craniocaudal landmark) and the Sylvian fissure (anterior–posterior landmark). For further statistical analysis, mean TMT values were used, including separately performed measurements of the right and the left TMT.

In case of previous interventions that may have affected the thickness of the temporal muscle (e.g., muscle edema or atrophy due to previous craniotomy or radiation therapy), the measurement of this side was excluded from further analysis.

### 4.3. Statistical Analysis

Statistical computations were performed using IBM SPSS Statistics for Windows version 26.0 and R version 3.6.1.

Metric data are described using mean and range (minimum, maximum). Categorical data are described by absolute frequencies and percentages. Previously, TMT cutoff values for males and females were determined with regard to the diagnosis of sarcopenia [19]. These sex-related TMT cutoff values were used to group patients into “below” and “above” the TMT cutoff groups. A log-rank test was performed and illustrated by a Kaplan–Meier plot that separated the TMT cutoff groups. Its association with overall survival in patients with PCNSL was tested using a univariate Cox model. To evaluate whether the effect of TMT on overall survival was confounded by other important prognostic variables (sex, age at time of diagnosis, deep brain involvement of the PCNSL lesions, ECOG performance status, immunodeficiency, and methotrexate-based chemotherapy), a multivariable Cox regression analysis was performed. The significance of the results was determined as a *p*-value < 0.05.

## 5. Conclusions

To summarize, TMT serves as an objective independent prognostic parameter in patients with PCNSL. The assessment of TMT on MR images in the routine clinical setting in PCNSL at the time of diagnosis may aid in treatment optimization and treatment decisions, as well as additional patient stratification for clinical trials, as it provides an objective evaluation of a frail patient population.

## Figures and Tables

**Table 1:** Overview of patient characteristics.

	*n*
Gender	
Male (%)	66 (52)
Female (%)	62 (48)
Mean age at time of diagnosis, years (range)	62.7 (23–84)
Immunodeficiency Deep brain involvement	10 (8%)
Yes (%)	41 (32%)
No (%)	87 (32%)
Median ECOG performance status * (range)	1 (0–4)
Methotrexate-based chemotherapy (%)	95 (74.2)
Mean overall survival, months (range)	31.9 (0–136)
Mean TMT **, mm (range)	6.3 (1.9–10.5)

* ECOG = Eastern Cooperation Oncology Group; ** TMT = temporal muscle thickness.

## Data Availability

Data is contained within the article.
